# Supplementary material for: NASA GeneLab Platform Utilized for Biological Response to Space Radiation in Animal Models
Source: Cancers (Basel). 2020 Feb 7;12(2):381. doi: 10.3390/cancers12020381 (PMC7072278; doi:10.3390/cancers12020381)
Supplement: Supplementary file 1 [file cancers-12-00381-s001.zip › Supplemental Figure Captions.docx]

**Figure S1.** Principal component analysis (PCA) plots on GeneLab datasets (GLDS) using the Gene Set Enrichment Analysis (GSEA) nominal enrichment scores before (top row) and after (bottom row) auto-annotation using Cytoscape’s enrichment map software. GSEA genesets used were as follows: (**a & b**) Gene Ontology (GO) terms; (**c & d**) C2 curated terms; and (**e & f**) C6 oncogenic signature terms. Each data point is represented by experimental radiation dose (color) or tissue type (shape).

**Figure S2.** t-Distributed Stochastic Neighbor Embedding (tSNE) plots on GeneLab datasets (GLDS) using the Gene Set Enrichment Analysis (GSEA) nominal enrichment scores before (top row) and after (bottom row) auto-annotation using Cytoscape’s enrichment map software. GSEA genesets used were as follows: (**a & b**) Gene Ontology (GO) terms; (**c & d**) C2 curated terms; and (**e & f**) C6 oncogenic signature terms. Each data point is represented by experimental radiation dose (color) or dose type (shape).

**Figure S3.** t-Distributed Stochastic Neighbor Embedding (tSNE) plots on GeneLab datasets (GLDS) using the Gene Set Enrichment Analysis (GSEA) nominal enrichment scores before (top row) and after (bottom row) auto-annotation using Cytoscape’s enrichment map software. GSEA genesets used were as follows: (**a & b**) Gene Ontology (GO) terms; (**c & d**) C2 curated terms; and (**e & f**) C6 oncogenic signature terms. Each data point is represented by experimental radiation dose (color) or tissue type (shape).

**Figure S4.** Analysis of all GeneLab Datasets utilized for direct comparisons with Gene Ontology (GO) GSEA analysis with FDR < 0.05 for 3 datasets. Heatmap with k-mean clustering for the specific GO pathways. Nine specific pathways where found to be clustered together through k-mean clustering. The specific 9 clusters are also shown.

**Figure S5.** Analysis of all GeneLab Datasets utilized for direct comparisons with Gene Ontology (GO) GSEA analysis with FDR < 0.05 for 3 datasets for cluster 2. Heatmap with k-mean clustering for the specific GO pathways. Four specific pathways where found to be sub-clustered together through k-mean clustering. The specific 4 sub-clusters are also shown.

**Figure S6.** Analysis of all GeneLab Datasets utilized for direct comparisons with Gene Ontology (GO) GSEA analysis with FDR < 0.05 for 3 datasets for cluster 3. Heatmap with k-mean clustering for the specific GO pathways. Nine specific pathways where found to be sub-clustered together through k-mean clustering. The specific 9 sub-clusters are also shown.

**Figure S7.** Scatter plots comparing the Normalized Enrichment Scores (NES) to Dose in mGy and fits with a Generalized Additive Model (GAM) on each cluster (represented in **Figure S4**). Each panel represents one of the clusters and the color-coded lines represent GAM fits done on each GO annotated term in the cluster. The circles in the plots represent the gene sets and the pink shade around the GAM fits represent the standard error.

**Figure S8.** Scatter plots comparing the Normalized Enrichment Scores (NES) to Dose in mGy and fits with a Generalized Additive Model (GAM) on each cluster (represented in **Figure S5**). Each panel represents one of the clusters and the color-coded lines represent GAM fits done on each GO annotated term in the cluster. The circles in the plots represent the gene sets and the pink shade around the GAM fits represent the standard error

**Figure S9.** Scatter plots comparing the Normalized Enrichment Scores (NES) to Dose in mGy and fits with a Generalized Additive Model (GAM) on each cluster (represented in **Figure S5**). Each panel represents one of the clusters and the color-coded lines represent GAM fits done on each GO annotated term in the cluster. The circles in the plots represent the gene sets and the pink shade around the GAM fits represent the standard error

**Figure S10.** Analysis of all GeneLab Datasets utilized for direct comparisons with C2 curated gene sets GSEA analysis with FDR < 0.05 for 3 datasets. Heatmap with k-mean clustering for the specific C2 pathways. Nine specific pathways where found to be clustered together through k-mean clustering. The specific 9 clusters are also shown.

**Figure S11.** Analysis of all GeneLab Datasets utilized for direct comparisons with C2 curated gene sets GSEA analysis with FDR < 0.05 for 3 datasets for cluster 1. Heatmap with k-mean clustering for the specific C2 pathways. 15 specific pathways where found to be sub-clustered together through k-mean clustering. The specific 15 sub-clusters are also shown.

**Figure S12.** Analysis of all GeneLab Datasets utilized for direct comparisons with C2 curated gene sets GSEA analysis with FDR < 0.05 for 3 datasets for cluster 2. Heatmap with k-mean clustering for the specific C2 pathways. 15 specific pathways where found to be sub-clustered together through k-mean clustering. The specific 15 sub-clusters are also shown.

**Figure S13.** Analysis of all GeneLab Datasets utilized for direct comparisons with C2 curated gene sets GSEA analysis with FDR < 0.05 for 3 datasets for cluster 3. Heatmap with k-mean clustering for the specific C2 pathways. 15 specific pathways where found to be sub-clustered together through k-mean clustering. The specific 15 sub-clusters are also shown.

**Figure S14.** Scatter plots comparing the Normalized Enrichment Scores (NES) to Dose in mGy and fits with a Generalized Additive Model (GAM) on each cluster (represented in **Figure S10**). Each panel represents one of the clusters and the color-coded lines represent GAM fits done on each GO annotated term in the cluster. The circles in the plots represent the gene sets and the pink shade around the GAM fits represent the standard error.

**Figure S15.** Scatter plots comparing the Normalized Enrichment Scores (NES) to Dose in mGy and fits with a Generalized Additive Model (GAM) on each cluster (represented in **Figure S11**). Each panel represents one of the clusters and the color-coded lines represent GAM fits done on each GO annotated term in the cluster. The circles in the plots represent the gene sets and the pink shade around the GAM fits represent the standard error

**Figure S16.** Scatter plots comparing the Normalized Enrichment Scores (NES) to Dose in mGy and fits with a Generalized Additive Model (GAM) on each cluster (represented in **Figure S12**). Each panel represents one of the clusters and the color-coded lines represent GAM fits done on each GO annotated term in the cluster. The circles in the plots represent the gene sets and the pink shade around the GAM fits represent the standard error

**Figure S17.** Scatter plots comparing the Normalized Enrichment Scores (NES) to Dose in mGy and fits with a Generalized Additive Model (GAM) on each cluster (represented in **Figure S13**). Each panel represents one of the clusters and the color-coded lines represent GAM fits done on each GO annotated term in the cluster. The circles in the plots represent the gene sets and the pink shade around the GAM fits represent the standard error

**Figure S18.** Analysis of all GeneLab Datasets utilized for direct comparisons with C6 Oncogenic Signature GSEA analysis with FDR < 0.05 for 3 datasets. Heatmap with k-mean clustering for the specific C6 pathways. Nine specific pathways where found to be clustered together through k-mean clustering. The specific 9 clusters are also shown.

**Figure S19.** Analysis of all GeneLab Datasets utilized for direct comparisons with C6 Oncogenic Signature GSEA analysis with FDR < 0.05 for 3 datasets for cluster 1. Heatmap with k-mean clustering for the specific C6 pathways. Nice specific pathways where found to be sub-clustered together through k-mean clustering. The specific 9 sub-clusters are also shown.

**Figure S20.** Scatter plots comparing the Normalized Enrichment Scores (NES) to Dose in mGy and fits with a Generalized Additive Model (GAM) on each cluster (represented in **Figure S18**). Each panel represents one of the clusters and the color-coded lines represent GAM fits done on each GO annotated term in the cluster. The circles in the plots represent the gene sets and the pink shade around the GAM fits represent the standard error.

**Figure S20.** Scatter plots comparing the Normalized Enrichment Scores (NES) to Dose in mGy and fits with a Generalized Additive Model (GAM) on each cluster (represented in **Figure S19**). Each panel represents one of the clusters and the color-coded lines represent GAM fits done on each GO annotated term in the cluster. The circles in the plots represent the gene sets and the pink shade around the GAM fits represent the standard error
