# Supplementary material for: NASA GeneLab Platform Utilized for Biological Response to Space Radiation in Animal Models
Source: Cancers (Basel). 2020 Feb 7;12(2):381. doi: 10.3390/cancers12020381 (PMC7072278; doi:10.3390/cancers12020381)
Supplement: Supplementary file 1 [file cancers-12-00381-s001.zip › Table S1-Ground Data table-02.docx]

**Table S1**. Doses and dose rates for ground-based exposures at the NASA Space Radiation Laboratory (NSRL) at Brookhaven National Laboratory. Dose rates for some studies were not available as indicated in the table by NA. Uncertainties in dose and dose rate are less than 2% [1]

| **GLDS** | **Radiation type** | **Particle charge** | **Particle mass** | **Energy (MeV/u (ions)) (MeV (gamma)) (kV (x-rays))** | **Particle LET (keV/μm)** | **Absorbed Radiation Dose Rate(s) (cGy/min)** | **Absorbed Radiation Dose(s)**  **(cGy)** |
| --- | --- | --- | --- | --- | --- | --- | --- |
| 73 | Fe | 26 | 56 | 1000 | 150 | NA | 0 |
| 73 | Fe | 26 | 56 | 1000 | 150 | NA | 50 |
| 73 | Fe | 26 | 56 | 1000 | 150 | NA | 100 |
| 73 | gamma | -- | -- | 0.6617 | -- | NA | 0 |
| 73 | gamma | -- | -- | 0.6617 | -- | NA | 100 |
| 73 | gamma | -- | -- | 0.6617 | -- | NA | 300 |
| 73 | Si | 14 | 28 | 290 | 44 | NA | 0 |
| 73 | Si | 14 | 28 | 290 | 44 | NA | 50 |
| 73 | Si | 14 | 28 | 290 | 44 | NA | 100 |
| 80 | gamma | -- | -- | 0.6617 | -- | NA | 0 |
| 80 | gamma | -- | -- | 0.6617 | -- | NA | 5 |
| 80 | gamma | -- | -- | 0.6617 | -- | NA | 10 |
| 80 | gamma | -- | -- | 0.6617 | -- | NA | 50 |
| 80 | gamma | -- | -- | 0.6617 | -- | NA | 70 |
| 80 | gamma | -- | -- | 0.6617 | -- | NA | 100 |
| 80 | gamma | -- | -- | 0.6617 | -- | NA | 200 |
| 80 | x-ray | -- | -- | 6000 | -- | NA | 3-200 |
| 80 | Si | 14 | 28 | 350 | 64 | NA | 0 |
| 80 | Si | 14 | 28 | 350 | 64 | NA | 10 |
| 80 | Si | 14 | 28 | 350 | 64 | NA | 20 |
| 80 | Si | 14 | 28 | 350 | 64 | NA | 50 |
| 80 | Si | 14 | 28 | 350 | 64 | NA | 100 |
| 80 | Si | 14 | 28 | 350 | 64 | NA | 200 |
| 80 | Fe | 26 | 56 | 600 | 190 | NA | NA |
| 109 | Fe | 26 | 56 | 1000 | 150 | 5 | 15 |
| 117 | p | 1 | 1 | 1000 | 0.24 | 17 | 90 |

1.	La Tessa, C.; Sivertz, M.; Chiang, I.H.; Lowenstein, D.; Rusek, A. Overview of the NASA space radiation laboratory. *Life Sci Space Res (Amst)* **2016**, *11*, 18-23, doi:10.1016/j.lssr.2016.10.002.
